# The Role of Parental Perfectionism and Child Temperament in the Intergenerational Transmission of Perfectionism: A Pilot Study

**DOI:** 10.3390/children12111452

**Published:** 2025-10-25

**Authors:** Diana Oliveira, Carolina Martins, Luís Faísca, Marta Brás, Cristina Nunes, Cláudia Carmo

**Affiliations:** 1 University Research Centre in Psychology (CUIP), Universidade do Algarve, Campus de Gambelas, 8005-139 Faro, Portugal; dianafernandesoliveira@gmail.com (D.O.); lfaisca@ualg.pt (L.F.); mbras@ualg.pt (M.B.); csnunes@ualg.pt (C.N.); 2 Psychology Service (SP), Faculdade de Ciências Humanas e Sociais, Universidade do Algarve, Campus de Gambelas, 8005-139 Faro, Portugal; cnmartins@ualg.pt

**Keywords:** child perfectionism, parental perfectionism, parental behaviours, intergenerational transmission, child temperament

## Abstract

**Background/Objectives:** Perfectionism is a personality trait characterised by the setting of extremely high and unrealistic personal standards, accompanied by critical self-evaluations. The literature indicates that perfectionism may develop as a learned behaviour, shaped by parent–child interactions, highlighting the influence of parental, individual and environmental factors. This quantitative study examines how parental perfectionism/practices and child temperament contribute to early perfectionism. **Methods:** The sample comprised 32 first-grade children (9 girls) from Faro district, aged between five and seven, and their parental figures. Parental perfectionism was assessed using self-report questionnaires, while children’s characteristics were evaluated through a combination of parent-report measures, direct observation, and interview-based methods. **Results:** Children self-rated higher perfectionism than parents attributed, with modest cross-informant agreement for socially prescribed and negligible agreement for Self-Oriented Perfectionism. Direct parent–child associations were small and method-dependent. Coercive/intrusive parenting corresponded to higher child Socially Prescribed Perfectionism, with convergence between observed intrusiveness and self-reported coercive practices. Temperament showed modest, patterned covariation with parenting and child perfectionism. Notably, Effortful Control attenuated the association between parental and child Socially Prescribed Perfectionism, whereas Surgency/Extraversion and Negative Affect did not; no temperament dimension moderated Self-Oriented Perfectionism. **Conclusions:** Findings indicate a complex interplay between dispositional and environmental factors in early childhood and underscore the value of multi-informant, multi-method assessment. As a pilot study, these findings provide initial insights into the intergenerational transmission of perfectionism in small children and serve as a basis for generating hypotheses and guiding future research, emphasising longitudinal designs and diverse samples to strengthen validity and clarify intergenerational processes.

## 1. Introduction

Perfectionism is a personality trait characterised by the setting of extremely high and unrealistic personal standards, accompanied by critical self-evaluations [1,2]. It typically involves rigid adherence to personal standards, a disproportionate significance to their attainment, marked presence of dichotomous thinking, a tendency to overgeneralise failures and an intense fear of failure [3,4,5,6].

Hewitt and Flett [7] conceptualised perfectionism as a multidimensional construct which includes three different dimensions: Self-Oriented Perfectionism (SOP), referring to the tendency of an individual to set excessively high personal standards for oneself; Socially Prescribed Perfectionism (SPP), involving the perception that others demand and expect perfection from oneself; and Other-Oriented Perfectionism (OOP), defined as the imposition of unrealistic, rigidly high standards on other people. This conception remains relevant, with evidence showing that the three dimensions are associated with a range of psychological disorders, as well as emotional, behavioural, and social problems in adults, adolescents, and children [8]. Thus, perfectionism is regarded as a transdiagnostic risk factor contributing to the onset of various psychological disorders [9].

Given its negative impact, researchers have increasingly investigated the contributing factors to the development of perfectionism, with several etiological models identifying childhood and adolescence as critical periods for its emergence. For instance, the Integrative Model of the Development of Perfectionism [2] emphasises multiple developmental pathways for the emergence of this trait, and distinguishes three broad categories of factors: parental factors (e.g., parenting practices and styles, parental personality, and goals), external environmental factors (e.g., culture, teachers, peers, occupation), and child-specific factors (e.g., attachment style and temperament).

Moreover, in a recent review of the developmental models of perfectionism, Flett and Hewitt [10] outlined a range of factors and associated processes that influence whether perfectionistic standards and external pressures are internalised and transformed into elevated Self-Oriented Perfectionism. These include both external and internal influences, such as the child’s receptiveness to socialisation and a strong desire to gain approval from others; exposure to a self-oriented perfectionist role model and a wish to imitate this individual; growing up in a family context that places high value on achievement and meeting high standards; possessing abilities in at least one domain where perfection is seen as attainable; and having a temperament marked by intense persistence and a certain degree of fearfulness.

The Pediatric Model of Perfectionism in Anxiety and Depression Disorders [11] also posits that a myriad of parental factors, such as psychopathology, perfectionism, and parenting behaviours, may contribute to the intergenerational transmission of perfectionism, shaping the expression of genetically inherited vulnerabilities in the child. This model also emphasises a multidirectional interaction between child temperament and perfectionism and parental factors, suggesting that these elements dynamically influence one another throughout development.

Thus, it can be concluded that the literature identifies not only parental influence but also child temperament as potential precursors in the development of perfectionism, e.g., [12,13,14].

### 1.1. Parental Influence

Empirical evidence suggests that perfectionism may stem from familial influences, particularly from perceived family perfectionism [15], parenting perceptions (e.g., perceived parental criticism and parental expectations [16]) and actual parenting practices (e.g., parenting styles [17]). Specifically, perfectionism often arises in environments marked by perceived parental disapproval, elevated expectations, and persistent criticism from caregivers [18], where children typically receive limited positive feedback, guidance, or affection [19,20]. In such contexts, children are more likely to develop perfectionistic tendencies not only when parents directly promote perfectionism—by imposing demanding standards [20] with low warmth [17] or employing harsh criticism as a disciplinary strategy in response to unmet expectations—but also when parents indirectly transmit perfectionism by modelling their own perfectionistic goals and standards [9,20]. Thus, parental perfectionism appears to significantly shape parenting behaviours that foster perfectionism in children [21].

Although empirical evidence supports the association between parental and child perfectionism, e.g., [9,22], research into the intergenerational transmission of perfectionism is relatively recent [21]. To date, no studies have specifically examined the origins of perfectionism in children younger than seven, with existing research focusing primarily on children aged seven and older [23]. Indeed, research on the etiological factors of perfectionism has relied primarily on adolescent and university samples, often employing correlational designs and self-report measures [23]. This highlights the need for studies that investigate perfectionism in younger children, while they are still living with their parents, incorporating observational methods in parent–child interaction contexts. Such approaches would enable a more comprehensive understanding of the developmental and processes involved in the emergence and maintenance of perfectionism in early childhood.

Considering the common practice of aggregating mothers and fathers in developmental research, evidence suggests that perceived expectations and criticism can be experienced and reported differently across caregivers, and cross-informant agreement is typically modest [24,25]. Meta-analytic and narrative reviews further indicate small but reliable mother–father discrepancies in ratings of child behaviour and, in some cases, differential associations of maternal versus paternal practices with youth outcomes; however, the evidence is mixed and often based on samples with a predominance of mothers [26].

### 1.2. Child Temperament

Child temperament refers to an innate and stable pattern of mood and behaviour that reflects constitutional differences in emotional, motor, and attentional reactivity [27,28] and determines not only the child’s behavioural style but also how they experience and respond to the world around them [21,29,30]. While temperament is primarily rooted in genetic factors [31,32], it is also shaped by central nervous system maturation, developmental processes, and learning experiences [33,34]. Temperament plays a foundational role in early development, as it contributes to the emergence of personality structures in childhood [33].

One of the most widely adopted frameworks for understanding child temperament is Rothbart’s psychobiological model [29], which defines temperament as constitutionally based individual differences in reactivity (i.e., the excitability and responsiveness of behavioural and physiological systems) and self-regulation (i.e., processes that modulate this reactivity) [35]. This model distinguishes two dimensions of the reactivity system—Extraversion and Negative Affect—and one dimension of the self-regulation system—Effortful Control. Extraversion is expressed through high activity levels, restlessness in social situations, preference for intense stimulation (i.e., risk-taking), and quick response tendencies [30]. In the early stages of development, Negative Affect is characterised by primitive forms of distress and irritability that later evolve into fear and frustration [30]. Effortful Control involves Inhibitory Control, the expression of enjoyment in low-intensity activities, and sustained focused attention [36,37,38]. Extreme levels of these temperament traits may increase vulnerability to psychopathology [31]. Longitudinal studies exploring stability and change in temperament traits have shown moderate continuity between childhood temperament and adult personality traits, e.g., [39].

Given that etiological models consider temperament as a key precursor to the development of perfectionism [20], a growing body of research has examined the relationship between the two constructs, e.g., [12,31,40]. For example, Walton et al. [17] found that personality traits were stronger predictors than parenting for both adaptive and maladaptive perfectionism dimensions, supporting the notion that perfectionistic tendencies are, at least in part, rooted in dispositional characteristics such as temperament. Furthermore, temperament may help explain the heterogeneity observed among individuals with perfectionistic traits [14].

Indeed, several studies conducted with child and adolescent samples suggest that specific temperamental features (e.g., anxious or inhibited temperament, higher levels of perseveration) may increase the likelihood of developing maladaptive forms of perfectionism [13].

Affrunti et al. [12], in a study involving children aged 7 to 13, found that inhibited temperament predicted maladaptive increases in both Socially Prescribed Perfectionism (SPP) and Self-Oriented Perfectionism (SOP), with these effects being exacerbated by parenting behaviours or peer interactions.

Similarly, Oros et al. [18], in a study with children aged 8 to 12, reported that neurotic temperamental traits combined with excessive parental performance demands may create a developmental context conducive to perfectionism.

In contrast, a longitudinal study by Hong et al. [40], conducted with seven-year-old children, found that most of the temperament dimensions under investigation (namely, Negative Affectivity and Effortful Control) showed limited power to predict the development of maladaptive perfectionism. Notably, child surgency was the only significant predictor for SPP trajectories. These results leave the role of temperament in the development of perfectionism still open to debate.

Over the past two decades, developmental research on perfectionism has advanced significantly, leading to a wide range of meaningful insights [10]. Evidence derived from studies examining developmental trajectories highlight the possible contributing role of factors such as parental intrusiveness and negative parenting [40], perceived parental psychological control, limited parental autonomy support and low parental responsiveness [38], and child temperament traits such as high surgency [40]. However, findings also reveal the intricate and multifaceted nature of perfectionism development, indicating that this area warrants ongoing empirical attention [41].

Based on the aforementioned, one might reasonably infer that parental perfectionism may influence specific parenting practices, such as intrusiveness, critical feedback, or performance-oriented demands, which in turn interact with child temperament characteristics—including shyness, Effortful Control, and emotional reactivity—to shape perfectionistic tendencies. The interplay between these dispositional and environmental factors is dynamic and bidirectional, meaning that children’s traits may elicit particular parenting responses, while parental behaviours can amplify or mitigate emerging perfectionistic patterns. Clarifying these mechanisms is essential for understanding the intergenerational transmission of perfectionism and for informing targeted interventions.

This conceptualisation aligns with the Pediatric Model of Perfectionism [11], which emphasises the role of parent–child dynamics in shaping perfectionistic traits, and with the Integrative Model of Perfectionism Development [2], which underscores the combined influence of temperament and environmental factors. These frameworks directly informed our hypotheses that (a) parental perfectionism and parenting practices would be associated with child perfectionism and (b) child temperament might moderate these associations, and guided the choice of a multi-method, multi-informant design.

### 1.3. The Current Study

This cross-sectional, exploratory, quantitative study aims to examine the extent to which parental factors (namely, parenting practices and parental perfectionism) and child temperament are associated with children’s perfectionism, in line with established explanatory models, using a multi-method, multi-informant assessment battery. To this end, children were assessed using observational measures of perfectionism and parental intrusive behaviour during copy and interactions tasks (e.g., mother–child, father–child), thereby minimising bias commonly associated with self-report and parent-report measures and enabling the identification of specific parental factors that may influence the development of perfectionism in children.

In line with this overall goal, the following specific objectives were defined: (1) to characterise the levels of child perfectionism using a multi-method approach, exploring differences across distinct measures of child perfectionism (namely, parent-reported, child self-reported and observational); (2) to explore the relationship between child perfectionism and parental factors, including parental perfectionism, general parenting behaviours, and specific parental behaviours (e.g., intrusiveness and encouragement/criticism) observed during a parent–child interaction task; (3) to examine the association between child temperament and child perfectionism; and (4) to test whether the main dimensions of child temperament (Surgency/Extraversion, Negative Affect, Effortful Control) moderate the association between parental and child perfectionism (SOP and SPP).

The study draws on multiple sources of information, including self- and parent-report data, as well as direct observational measures involving both parents and children. This multi-method approach enables a comprehensive assessment of child and parental perfectionism, parenting practices, and the dynamics underlying the intergenerational transmission of perfectionism. Given its exploratory nature, this study aims to provide preliminary insights that can inform future large-scale investigations on the development of perfectionism in early childhood.

## 2. Materials and Methods

### 2.1. Participants

Children from three primary schools in the district of Faro (Portugal) were recruited for this study. The final sample consisted of 32 children enrolled in the first year of primary education, aged between five and seven years (*M* = 6.03; *SD* = 0.31), including 9 girls (28.1%). One parent of each child also participated (30 mothers and 2 fathers), reporting an average age of 39.7 years (*SD* = 3.36), with 71.9% having a university-level education. Participants (either the child or parental figure) who lacked a sufficient command of the Portuguese language were excluded from the study.

### 2.2. Measures

#### 2.2.1. Child Protocol

##### Child and Adolescent Perfectionism Scale (CAPS)—Adapted Interview

Child perfectionism was assessed using an adapted interview version [42] of the Child and Adolescent Perfectionism Scale (CAPS) [43]. This interview measures two dimensions of perfectionism: Socially Prescribed Perfectionism (SPP; α = 0.77) and Self-Oriented Perfectionism (SOP; α = 0.69). The items are first assessed using a “yes” or “no” response format (e.g., “Do you try to be perfect in everything you do?”), followed by an evaluation of the level of certainty on a four-point scale (1—no, it is not true; 4—yes, it is completely true). The interview was translated into Portuguese using a standard forward–review–back-translation workflow by bilingual psychologists, and subsequently reviewed by an English language expert, who conducted a back-translation to verify accuracy and equivalence. Prior to data collection, we conducted pilot cognitive checks with first-grade children to ensure semantic and developmental appropriateness. The subscales demonstrated good to satisfactory internal consistency in our sample (SPP α = 0.79 and SOP α = 0.67).

##### Figure Copy Task

In the Figure Copying Task, children are asked to reproduce seven simple geometric figures with maximum accuracy, one at the time, allowing for the observation of self-oriented perfectionistic behaviours [23]. They are allowed to use a ruler and an eraser and have one minute to complete the entire task. Two investigators were present in the room to monitor timing and standardised instructions and did not interact with participants beyond scripted prompts.

In addition to assessing copy accuracy, children’s behaviour during the task was also recorded using the Child Behaviour Observation Grid [23]. Perfectionism levels were coded into five categories based on anchor points for representative behaviours outlined in the original coding manual (e.g., repeated or excessive checking and a slower pace of task completion to avoid mistakes): “Extremely Perfectionistic”, “Very Perfectionistic”, “Moderately Perfectionistic”, “Slightly Perfectionistic”, or “Not Perfectionistic”. Additionally, the frequency of four specific correction and verification actions was recorded: using the ruler for drawing, using the ruler for measuring, using the eraser, and repeatedly inspecting the stimulus book. Both researchers independently coded 18.75% of the behaviour records, achieving an intra-class correlation of 0.89. The remaining records were coded independently by one of the investigators.

##### Interaction Task

The interaction task intends to measure parental intrusive behaviour. The task is adapted from Hong et al. [40], involving a mother/father–child dyad collaborating to solve puzzles using the *Rush Hour* tabletop game by ThinkFun (Alexandria, VA, USA). The child is encouraged to complete as many levels as possible within a 10 min period and may receive assistance from the parent as needed. Researchers remain present during the task to monitor the session. Parent–child interactions were recorded using an observation grid adapted from the original study, which classified parental behaviour into four levels of intrusiveness: “Non-Intrusive Type,” “Mildly Intrusive Type,” “Moderately Intrusive Type,” or “Extremely Intrusive Type”. In this framework, non-intrusive behaviours involve supporting the child’s autonomy without direct interference; mildly intrusive behaviours include gentle guidance or suggestions; moderately intrusive behaviours involve directing the child’s actions or decisions; and extremely intrusive behaviours are characterised by overt control, overriding the child’s choices, or completing the task on their behalf. These levels were assigned weights from 1 to 4, enabling the calculation of a global intrusiveness score for the task (*IT score*), based on the weighted frequency of the parents’ coded behaviours. An additional score, corresponding to the percentage of behaviours classified as moderately or extremely intrusive, was also computed (MEIT score).

#### 2.2.2. Parent Protocol

##### Sociodemographic Questionnaire

Sociodemographic data were collected using a Sociodemographic Questionnaire (SDQ), assessing participants’ personal, social, and demographic information (e.g., age, place of birth, nationality, marital status, educational background, and occupation, and information about the child, namely date of birth, gender, place of birth, nationality, and relevant life events during the past year).

##### Multidimensional Perfectionism Scale

Hewitt and Flett’s Multidimensional Perfectionism Scale (HMPS) [7] consist of 45 items scored on a 7-point Likert scale (1—Strongly Disagree; 7—Strongly Agree), distinguishing three dimensions of perfectionism: Self-Oriented Perfectionism (SOP, e.g., “I worry about getting a perfect result in everything”), Socially Prescribed Perfectionism (SPP, e.g., “I feel that other people expect too much from me”), and Other-Oriented Perfectionism (OOP, e.g., “I expect a lot from the people who are important to me”). The Portuguese version of the HMPS [44] used in this study shows adequate psychometric properties (SPP: α = 0.83; SOP: α = 0.89; OOP: α = 0.69). In the present study, only the SPP and SOP subscales were analysed, both also showing good internal consistency (α = 0.74 and α = 0.78, respectively).

##### Child and Adolescent Perfectionism Scale

The Child and Adolescent Perfectionism Scale (CAPS) [43] evaluates perfectionism in children and adolescents using 22 items rated on a 5-point scale (1, “completely false”, to 5—“completely true”), distributed across two subscales: SOP (Self-Oriented Perfectionism, e.g., “My child tries to be perfect in everything he/she does”) and SPP (Socially Prescribed Perfectionism, e.g., “My child feels that there are people in his/her life who expect him/her to be perfect”).

This study used the CAPS Portuguese version [45] (internal consistency for the total scale: α = 0.81), which was adapted for use as a parental-report measure of children’s perfectionism. With the present sample, the full scale demonstrated very good internal consistency (α = 0.84), as did the subscale SOP (α = 0.83); the subscale SPP exhibited satisfactory internal consistency (α = 0.76).

##### Parental Behaviour Scale

The Parental Behaviour Scale (PBS) [46] consists of 45 items scored on a 5-point rating scale (from “never” to “always”) and assesses parents’ behaviours towards their children. It is divided into two global subscales, Positive Parenting (PP) and Coercive Parenting (CP), encompassing nine dimensions. The PP subscale reflects supportive and nurturing practices, such as Positive Reinforcement (e.g., “I make time to listen to my child”), Monitoring (e.g., “I keep up to date about the friends my children go out with”), Rules (e.g., “I teach my children to obey rules”), and Autonomy (e.g., “I teach my child to solve his/her own problems”); the CP subscale captures controlling and punitive strategies, including Discipline (e.g., “When my child does not behave, I give him/her a task as a punishment”), Inconsistent Discipline (e.g., When I punish my children, I sometimes let them off the punishment early”), Harsh Punishment (e.g., “I give my children a smack when we argue”), Ignoring (e.g., “When my child does something that is not allowed, I give him/her an angry look and pretend he/she is not there”), and Material Rewarding (e.g., “When my children do their best, I allow something extra”). Validation studies [46] showed good reliability for the dimensions of Positive Reinforcement, Rules, Discipline, and Harsh Punishment (α ≥ 0.80), moderate reliability for Material Rewarding and Ignoring (α ≥ 0.70), and acceptable to low reliability for Monitoring, Inconsistent Discipline, and Autonomy (α between 0.52 and 0.68).

In the present study, the Portuguese version of the PBS [47] was used, which demonstrated very good reliability in our sample. The analysed dimensions generally showed satisfactory to excellent internal consistency (e.g., Harsh Punishment, α = 0.90; Rules, α = 0.85; Ignoring, α = 0.87; Positive Reinforcement, α = 0.78), apart from the Autonomy dimension, which showed low consistency (α = 0.54), likely due to the small number of items. The two global subscales also demonstrated excellent reliability (Positive Parenting, α = 0.79; Coercive Parenting, α = 0.90).

##### Children’s Behaviour Questionnaire

Children’s Behaviour Questionnaire—Short Form (CBQ-SF) [48] is a hetero-report instrument (i.e., completed by the primary caregiver), consisting of 94 items rated on a 7-point scale (1—“extremely untrue” to 7—“extremely true”), with an additional “not applicable” option for behaviours that parents consider not to occur in their child. In the present study, the Portuguese version of the instrument was used [49], which includes 15 subscales organised into three broad dimensions: Surgency/Extraversion, Negative Affect and Effortful Control.

Most subscales showed satisfactory internal consistency (e.g., Soothability/Falling Reactivity: α = 0.78 [e.g., “When upset, he/she feels better within a few minutes”]; Inhibitory Control, α = 0.76 [e.g., “Can wait to start an appealing activity when told to wait”]; Activity Level, α = 0.69 [e.g., “My child seems always in a hurry to move from one place to another”]), although some displayed low values (e.g., Anger/Frustration, α = 0.48 [e.g., “Throws tantrums when he/she doesn’t get what he/she wants”]; Sadness, α = 0.36 [e.g., “Tends to feel sad if family plans are changed or do not happen”]; Fear, α = 0.04 [e.g., “Is afraid of loud noises”]). Therefore, we limited our analysis to subscales with Cronbach’s *alpha* coefficient above 0.6. At the dimension level, Surgency/Extraversion (α = 0.77) and Effortful Control (α = 0.82) demonstrated satisfactory to very good internal consistency in the current study. The Negative Affect dimension (α = 0.61) exhibited relatively lower reliability, possibly be due to the heterogeneity of its items, as it encompasses distinct emotional responses such as fear, sadness, shyness and frustration.

### 2.3. Procedure and Statistical Analysis

Following approval by the Portuguese Ministry of Education and the Directors of the Executive Boards of each selected school (public and private institutions offering the 1st Cycle Basic Education in the Faro district), schools were contacted to assess their interest in participating in the study. Following institutional approval, class coordinators distributed invitations to legal guardians; interested parents contacted the research team and were scheduled for on-site sessions at the schools. Meetings were held to present and schedule the study. Informed consent was obtained from the legal guardians of participating children, in accordance with the guidelines of Law No. 67/98 of October 26 and the General Data Protection Regulation (EU) 2016/679 of the European Parliament.

The study complied with the Declaration of Helsinki and received ethics approval from the Scientific Commission of the Psychology and Educational Sciences Department, University of Algarve (Ref. EDOC/2018/23460, 10 September 2018). Given the involvement of young children, sessions were conducted on school premises during short, scheduled blocks, with the parent present on site, and procedures tailored to be developmentally appropriate; participation was voluntary and could be discontinued at any time without penalty.

Participants (parent–child dyads) were contacted via email to schedule a session which took place in a designated room within the school. Data collection consisted of phases: (1) child assessment (~15 min): individual administration of the CAPS—Adapted Interview and the Figure Copy Task; (2) parent–child interaction task (~10 min): during this task, parental and child behaviours were observed and coded; (3) parental questionnaires (~25 min): parents were given a paper-based protocol to complete at home, which include the Sociodemographic Questionnaire, HMPS [7], CAPS [41], PBS [44], and CBQ-SF [46]. This last step was conducted after the interaction task to minimise social desirability bias.

Finally, the data were organised and analysed using IBM SPSS Statistics software (version 24.0). Inferential analyses included paired-sample *t* tests and Pearson correlations to examine mean differences and associations, as well as hierarchical linear regression to test moderation effects. Despite the pilot sample size (*N* = 32), these parametric procedures were retained because they are widely used in small-sample developmental research and are generally robust to modest departures from normality at the group level. Sensitivity analyses using equivalent nonparametric methods (Wilcoxon signed-rank test and Spearman’s rho inference) yielded comparable results but are not reported here to avoid redundancy in the presentation of findings. For regression-based moderation analyses, results should be interpreted with caution, as small samples and non-normal variables can reduce statistical power and increase model instability. To address this, the analyses were complemented by robustness checks using bootstrapped confidence intervals for regression coefficients, which are reported in the Results section. Bootstrapped 95% confidence intervals (5000 samples) were based on bias-corrected estimates computed with PROCESS [50]. To privilege estimation over dichotomous inference, effect sizes (Cohen’s *d*, Pearson’s *r*) are reported alongside *p* values and findings are interpreted conservatively in light of sample size and measurement constraints.

## 3. Results

### 3.1. Analysis of Children’s Perfectionism Measures

The analysis of children’s behaviour during the Figure Copying Task indicated that most participants exhibited low levels of perfectionism (Table 1). This observational measure of children’s perfectionism was strongly and negatively correlated with the number of figures completed (Pearson’s correlation, *r* = −0.69, *p* < 0.001), and positively correlated with performance-related corrective behaviours, such as increased use of a ruler (*r* = 0.43, *p* = 0.013) and eraser (*r* = 0.59, *p* < 0.001) during the task.

The use of two measures of children’s perfectionism based on CAPS, one self-reported and one parent-reported, enabled a multi-informant assessment of this personality trait. While parents rated their children around the midpoint of the scale for both SOP and SPP, children rated themselves as considerably more perfectionistic in both dimensions, with large effect sizes for these differences (Cohen’s *d* ≥ 0.8). Despite this agreement discrepancy, consistency between informants varied: self- and parent-reported SOP showed a low, non-significant correlation (*r* = 0.14, *p* = 0.452), whereas self- and parent-reported SPP were moderately and significantly correlated (*r* = 0.47, *p* = 0.007; Table 2).

A detailed item-level analysis comparing the CAPS interview (child self-report) and the CAPS (parent report) revealed generally weak and non-significant correlations between directly corresponding items (see diagonal on Table 3). Most matched item pairs demonstrated little convergence, with coefficients ranging from slightly negative (*r* = −0.09) to slightly positive (*r* = 0.20). One notable exception emerged between Point 4 (child-reported SPP) and Item 5 (parent-reported SPP), where a moderate and statistically significant correlation was found (*r* = 0.44, *p* = 0.011), suggesting some agreement regarding the perception of external expectations placed on the child.

Beyond the directly corresponding items, several moderate to strong correlations emerged across dimensions, revealing a more nuanced relationship between child and parent perceptions of perfectionism. Notably, Point 3, which reflects Self-Oriented Perfectionism in the child interview, was positively associated with two parent-rated SPP items—Item 5 (*r* = 0.38, *p* = 0.031) and Item 8 (*r* = 0.48, *p* = 0.005). This suggests that children who report a strong drive to meet personal standards may be perceived by their parents as subject to external expectations, both from significant others and from the family context. A similar pattern was observed for Point 4 (also SPP, child version), which correlated strongly with Item 8 (*r* = 0.53, *p* = 0.002), again indicating some convergence between the child’s sense of social pressure and the parent’s recognition of family-driven perfectionistic demands.

Interestingly, these associations often crossed dimensions. While child- and parent-rated SOP showed little direct alignment, child-reported SOP items such as Point 3 were nonetheless significantly associated with parent-rated SPP items. This cross-dimensional pattern may suggest that children internalise external demands as self-imposed standards, or alternatively, that parents interpret their child’s high striving as resulting from outside pressure rather than internal motivation.

Correlations between performance on the Figure Copying Task and both self- and parent-reported measures of child perfectionism (Table 4) revealed only modest associations between observed and reported indices of the construct. Behaviourally observed perfectionism during the task correlated positively with self-reported SOP (*r* = 0.34, *p* = 0.045; see Table 4); correlations with SPP, whether self- or parent-reported, are also positive, but non-significant (0.22 ≤ *r* ≤ 0.26, *p* > 0.1). Furthermore, the number of figures completed showed only negligible, non-significant correlations with all questionnaire-based measures (−0.24 ≤ *r* ≤ 0.09, *p* > 0.2), suggesting that efficiency in task completion is largely independent of how perfectionism is perceived by either the child or their parents.

### 3.2. Analysis of the Influence of Parental Factors on Children’s Perfectionism

Parents completed the HMPS to assess their own levels of perfectionism, as well as the PBS, a self-reported measure of parenting behaviours in terms of Positive Parenting (PP) and Coercive Parenting (CP). Additionally, the interaction task provided an observational measure of parental intrusiveness while collaborating with their children on a performance task. Descriptive statistics and correlations among these parental measures are presented in Table 5.

In the interaction task, the most observed parental behaviours were classified as Mildly Intrusive Type (*M* = 48.0%, *SD* = 13.2%), characterised by verbal encouragement, and Non-Intrusive Type (*M* = 35.4%, *SD* = 19.7%), involving strategies such as open-ended questions without direct interference. Moderately intrusive behaviours (*M* = 14.4%, *SD* = 15.6%) and extremely intrusive behaviours (*M* = 2.3%, *SD* = 4.3%) were markedly less frequent, resulting in a low MEIT score (*M* = 16.7%). Behavioural patterns suggest that parents often combine two behavioural styles simultaneously—typically either the least intrusive or the most intrusive types. Overall, verbal support and encouragement strategies predominated, with minimal direct interference or excessive control over the child’s task. The average intrusiveness score (IT) is slightly below 2, indicating the prevalence of non-intrusive and mildly intrusive behaviours during the task.

Correlations between self-reported parental perfectionism (HMPS) and parenting behaviour (PBS) are small (−0.05 ≤ *r* ≤ 0.22), with one exception: a significant negative association between SPP and PP (*r* = −0.50, *p* = 0.004). Thus, while Coercive Parenting behaviours showed positive, albeit non-significant, correlations with these perfectionism dimensions, Positive Parenting behaviours were moderately and inversely associated with Socially Prescribed Perfectionism. Moreover, interaction task measures were strongly and positively correlated only with Coercive Parenting (*r* ≥ 0.54, *p* < 0.001), suggesting that greater observed intrusiveness during the task corresponded to higher self-reported use of controlling or punitive strategies. This convergence between observed and reported coercive behaviours supports the ecological validity of the interaction-based observational measure. By contrast, intrusiveness measures were only weakly and non-significantly correlated with SPP scores (*r* ~ 0.2, *p* > 0.1).

To examine the mutual influences between parental characteristics (perfectionism, parenting behaviours, and intrusiveness) and child perfectionism, correlations among these variables were computed (see Table 6).

The analysis of associations between parental perfectionism (HMPS) and the various measures of child perfectionism showed generally weak correlations (see Table 6), whether perfectionism was self-reported by children (−0.13 ≤ *r* ≤ 0.05), reported by parents (0.05 ≤ *r* ≤ 0.20), or assessed through observational measures (−0.06 ≤ *r* ≤ 0.01), suggesting no meaningful linear relationships with parental perfectionism dimensions.

Correlations between parenting behaviours (PBS) and child perfectionism were similarly negligible and non-significant for Positive Parenting (−0.10 ≤ *r* ≤ 0.09). Coercive Parenting, however, showed consistently positive, marginally significant associations with child-reported SPP (*r* = 0.30, *p* = 0.100) and parent-reported SPP (*r* = 0.31, *p* = 0.092).

Moderate positive correlations were found between intrusive parental behaviours (as assessed by the interaction task) and children’s SOP as reported by parents (MEIT: *r* = 0.36, *p* = 0.045; IT score: *r* = 0.33, *p* = 0.061). These intrusiveness measures also correlated positively with child-reported SPP (*r*~0.3) and parent-reported SPP (*r*~0.3), albeit not reaching significance (*p* < 0.08). Taken together, these findings suggest that higher levels of intrusive parenting are associated with increased SOP in children, at least from a parental perspective, whereas behavioural intrusiveness and Coercive Parenting are linked to higher SPP, particularly from the child’s perspective.

Interestingly, intrusiveness indicators (MEIT) from the interaction task were positively, though marginally, associated with productivity in the Figure Copying Task (*r* = 0.34, *p* = 0.061) and negatively associated with observational measures of child perfectionism (*r* = −0.26, *p* = 0.148). This pattern suggests that children’s task performance may be linked to the degree of parental intrusiveness during interaction, particularly in structured performance contexts.

### 3.3. Correlational Analysis Between Parental Behaviours/Perfectionism, Child Temperament and Child Perfectionism

The analysis of associations between child temperament dimensions (CBQ-SF) and perfectionism revealed predominantly small, non-significant correlations (–0.17 ≤ *r* ≤ 0.25; Table 7). For each dimension, one corresponding temperament subscale showing significant or marginally significant associations with child’s perfectionism is highlighted. Specifically, High-Intensity Pleasure (a Surgency/Extraversion subscale) correlated positively with parent-reported SOP (*r* = 0.40, *p* = 0.025), suggesting that children who derive strong enjoyment from situations involving high levels of stimulation, complexity, or novelty are perceived by parents as holding higher perfectionistic standards. Inhibitory Control (an Effortful Control scale) correlated negatively, although only marginally significantly, with parent-reported SOP (*r* = 0.31, *p* = 0.085), indicating that when children are described by parents as better able to regulate behaviour and suppress impulsive actions, parents also tend to be perceived that their perfectionistic behaviours as less driven by external pressure. Finally, Soothability /Falling Reactivity (a Negative Affect subscale, when inverted) correlated positively, albeit marginally significantly, with child-reported SPP (*r* = 0.30, *p* = 0.099), suggesting that children perceived by parents as able to calm down and recover more easily from distress are more likely to feel pressured to meet others’ expectations or to believe that acceptance and approval depend on being perfect.

The analysis of the relationship between parental behaviours/perfectionism and child temperament revealed several moderate associations (Table 8). Surgency/Extraversion showed moderate positive correlations with parents’ SPP (*r* = 0.36, *p* = 0.046) and both measures of parent intrusiveness (*r*~0.4, *p* < 0.03). Specifically, High-Intensity Pleasure, an Surgency/Extraversion subscale, correlated positively with parents’ SOP (*r* = 0.39, *p* = 0.027), Coercive Parenting (*r* = 0.38, *p* = 0.033) and intrusiveness (IT score: *r* = 0.37, *p* = 0.037; MEIT: *r* = 0.38, *p* = 0.031), suggesting that parents of highly extraverted children, who are motivated by high-intensity experiences, may hold stronger perfectionistic expectations for themselves and exhibit more controlling or intrusive behaviours. The Effortful Control dimension (and, specifically, the Inhibitory Control subscale) correlated positively with Positive Parenting (*r* = 0.36, *p* = 0.044; *r* = 0.35, *p* = 0.050, respectively), indicating that children with stronger self-regulation and attentional control tend to elicit more positive and supportive parenting behaviours. In contrast, the Negative Affect dimension showed no significant correlations with parental characteristics. The strongest correlation was positive with Coercive Parenting (*r* = 0.28, *p* = 0.118). Shyness (a Negative Affect subscale) showed a similar pattern, correlating higher with Coercive Parenting (*r* = 0.27, *p* = 0.133).

### 3.4. Moderation Analyses: The Role of Child Temperament in the Association Between Parental and Child Perfectionism

Separate hierarchical linear regression models were utilised to test whether each of the main dimensions of child temperament—Surgency/Extraversion, Negative Affect, and Effortful Control—moderated the association between parental and child perfectionism. In each model, parental perfectionism (centred) and the respective temperament dimension (centred) were entered in the first step, followed by the interaction term in the second step (Table 9).

For Self-Oriented Perfectionism (SOP), none of the child temperament dimensions showed a moderating effect: the inclusion of the interaction term explained less than 6% of additional variance (ΔR^2^ ≤ 0.056, *p* > 0.2). In contrast, for Socially Prescribed Perfectionism (SPP), Effortful Control exerted a significant negative moderation effect (R^2^ = 0.179, standardised β = −0.48, *p* = 0.020; bootstrapped 95% CI does not include zero). This finding suggests that higher levels Effortful Control in children weakened, and at higher levels, even reversed, the positive association between parental and child SPP. Neither Surgency/Extraversion nor Negative Affect significantly moderated the relationship between parental and child SPP (*p* ≥ 0.4).

## 4. Discussion

This study aimed to explore the relationship between parental perfectionism, parenting practices, child temperament and the manifestation of perfectionist traits in children in the first year of primary school. Despite growing interest in research on perfectionism—particularly its intergenerational transmission—gaps remain, especially concerning the role of parents and child temperament in the development of this trait. The results provide partial support for the selected theoretical models, highlighting the relevance of parenting practices and temperamental characteristics in the origin and development of childhood perfectionism. To our knowledge, this is one of the first studies to examine the intergenerational transmission of perfectionism in children younger than seven years old, using a multi-method approach that combines parent- and self-report data with observational assessments. This methodological contribution represents an important advancement in addressing limitations of previous research relying solely on questionnaires or adolescent samples, e.g., [21,23].

The combined analysis of self-reports and parent reports revealed only partial agreement between children’s and parents’ perceptions of childhood perfectionism. Parents tended to rate their children around the midpoint for both SOP and SPP, whereas children rated themselves as considerably more perfectionistic in both dimensions, with large effect sizes. This discrepancy suggests that parents may underestimate the intensity of their children’s internal demands, a pattern consistent with studies emphasising that subjective self-perception plays a critical role in the manifestation of perfectionistic tendencies [20,22]. At the item level, correlations revealed stronger convergence around Socially Prescribed Perfectionism, with the most significant agreement emerging between children’s and parents’ ratings of SPP items. Overall, these findings indicate that parents are more attuned to external pressures and expectations experienced by their children than to their self-imposed standards, highlighting a divergence in how different facets of perfectionism are mutually perceived. In the Figure Copying Task, higher levels of perfectionism were associated with lower productivity, as perfectionist children tend to prioritise accuracy over quantity. This finding supports previous results, e.g., [4,23], indicating that fear of failure can lead to functional hesitation, hindering task completion. Collectively, these findings underscore the potential divergence between behavioural manifestations of perfectionism in structured performance contexts and subjective perceptions of this trait.

In the family context, parental behaviours seem to shape children’s perfectionism in facet-specific ways. Coercive and intrusive parenting behaviours are linked to Socially Prescribed Perfectionism, suggesting that children perceive external pressures to meet expectations and gain approval. Conversely, parents who perceive their children as having high Self-Oriented Perfectionism (SOP) may also exhibit intrusive behaviours, although this does not appear to directly foster SOP in the child. Interestingly, Positive Parenting was not associated with any of the children’s perfectionism measures. These findings are consistent with Mitchell et al. [23], as parental behaviours emphasising precision and failure avoidance tend to be mirrored in children’s behaviours, raising their perfectionism levels.

Although most correlations between child temperament dimensions and perfectionism were small and non-significant, some patterns provide insight into the role of temperament in shaping perfectionistic tendencies. Children who experience High-Intensity Pleasure were perceived by parents as having higher Self-Oriented Perfectionism, suggesting that a propensity for intense engagement with stimulating or novel situations may amplify perfectionistic standards, at least from parents’ perspective. Conversely, higher Inhibitory Control is associated with lower perceptions of perfectionism, indicating that better self-regulation and emotional recovery may buffer against the development of perfectionistic tendencies. These trends align with prior findings suggesting that certain temperamental profiles may function as protective factors against the development of maladaptive perfectionism [12,13,18,40]. Overall, these findings point to a subtle interplay between dispositional emotional–attentional characteristics and the emergence of perfectionism in children.

Furthermore, moderate associations between child temperament and parental characteristics suggest a bidirectional influence in parent–child dynamics. Children high in Surgency/Extraversion, particularly those scoring high on High-Intensity Pleasure, were perceived by parents as having stronger Self-Oriented Perfectionism and tended to evoke more controlling or intrusive parenting, possibly reflecting attempts to channel their high-energy behaviours into structured tasks [12,17,40]. In contrast, children with higher Effortful Control, especially in inhibitory regulation, tended to elicit more positive and supportive parenting. Although non-significant, these results suggest that more inhibited children may experience greater parental control and even harsh discipline, possibly as a compensatory strategy to encourage task engagement. Overall, these findings indicate that children’s temperamental traits both influence and are influenced by parental behaviours, contributing to the development of perfectionistic tendencies.

Beyond examining direct associations, the present study also tested whether child temperament dimensions moderated the relationship between parental and child perfectionism. Of the three dimensions—Surgency/Extraversion, Negative Affect, and Effortful Control—only Effortful Control showed a statistically significant moderation effect, attenuating the influence of parents’ SPP on child SPP levels. This suggests that strong Effortful Control may buffer children from feeling pressured to meet external expectations, allowing them to maintain more autonomy in their performance standards despite parental pressures. In contrast, Surgency/Extraversion and Negative Affect did not emerge as significant moderators, indicating that these traits may play a lesser role in this dynamic, at least within the present sample. However, given the actual sample size, these null findings should be interpreted cautiously and verified in future studies using larger, more diverse samples. These results align with previous evidence that temperament may function not only as a direct correlate but also as a contextual factor in the intergenerational transmission of perfectionism and highlights the protective role of certain temperament traits in mitigating these tendencies.

Overall, the results reinforce the notion that childhood perfectionism emerges from a dynamic interaction between parental behaviours and the child’s dispositional characteristics. This claim is supported by most of the referenced studies in the literature, e.g., [22]. Controlling and demanding parenting styles appear to foster perfectionistic tendencies, particularly in socially evaluative contexts, while certain temperament traits may either amplify or mitigate these effects [12,13,18,40]. These results carry practical implications: interventions should encourage parents to create error-tolerant and supportive environments, while child-focused strategies aimed at strengthening emotional coping and cognitive flexibility could further support adaptive development [10,20]. Recognising the role of temperament as a potential moderator may inform the tailoring of intervention strategies [12,18,40].

## 5. Conclusions

The development of childhood perfectionism appears to be influenced by multiple factors, including attachment styles with parental figures, child temperament, perceived competence, and openness to socialisation. The literature shows that both parenting practices and sociocultural contexts, as well as individual child characteristics, are crucial in the emergence and maintenance of this trait. However, ambiguity remains regarding the specific weight of parental factors, which served as the main motivation for the present study.

This work aimed primarily to investigate the intergenerational transmission of perfectionism, focusing on observational measures in parent–child interaction contexts, complemented by both parent-report and self-report measures. The results suggest that childhood perfectionism can emerge at early ages—specifically before age five—and is significantly influenced by parental behaviours and attitudes. The use of observational methods proved valuable, as it reduced the risk of bias associated with social desirability often present in self-report-only assessments.

Overall, only partial agreement emerged between parent and child perceptions of perfectionism, with parental traits and behaviours—particularly perfectionistic expectations and intrusiveness—showing meaningful links to children’s perfectionism. Temperamental characteristics displayed both protective and risk associations, influencing how perfectionism manifests in children and interacts with parenting styles.

### 5.1. Significance and Implications of the Study

In summary, the findings reinforce the central role of parental behaviours and attitudes in shaping childhood perfectionism and highlight the potential moderating influence of child temperament. These results provide preliminary empirical support for theoretical models of perfectionism development [9] and may inform prevention and intervention strategies targeting families with young children.

### 5.2. Limitations

Among the study’s limitations, the small sample size is particularly noteworthy, as it restricted the use of more sophisticated statistical techniques to examine potential mediating and moderating effects of variables such as child sex, parenting behaviours, and specific temperament subdimensions. The reduced statistical power also meant that only moderate-to-large correlations (*r* ≥ 0.40) reached significance, while several potentially meaningful associations may have gone undetected. Moreover, the sample was drawn exclusively from a single geographic region in southern Portugal and consisted predominantly of highly educated parents, further limiting the generalisability of the findings. Because informants were predominantly mothers (*n* = 30; fathers *n* = 2), mother–father differences could not be examined, and findings should be interpreted accordingly.

Some measures, particularly the Material Rewarding subscale of the Parental Behaviour Scale and certain Children’s Behaviour Questionnaire subscales, exhibited low internal consistency, likely due to the small number of items and the challenges of assessing complex constructs in young children. Social desirability may also have influenced both child self-reports and observed behaviours. Additionally, the observational approach, although informative, carries the risk of reactivity effects, as parents and children may adjust their behaviour when aware of being observed.

### 5.3. Recommendations

Future research should involve larger and more diverse samples, including families from a wider range of educational and socioeconomic backgrounds, to enhance ecological validity and provide a more robust test of these mechanisms.

Future studies should prioritise psychometrically robust, developmentally appropriate, and multi-method approaches (e.g., behavioural assessments) to strengthen validity and reliability.

Although this study primarily focused on parental influences, prior literature also points to possible bidirectional effects, whereby children’s perfectionistic behaviour may shape parental practices [23]. Future research should explicitly examine these reciprocal dynamics using longitudinal designs capable of disentangling developmental trajectories, causal pathways and clarify intergenerational mechanisms underpinning the emergence of perfectionism in childhood.

Importantly, given the pilot and exploratory nature of the study, along with the relatively homogeneous sample, these findings should be interpreted with caution, and their generalisability is limited. Nevertheless, the study provides early evidence that perfectionism may emerge before age six and highlights the need for longitudinal, multi-informant, and multi-method research to explore the bidirectional dynamics between child perfectionism and parental behaviours, and to clarify when temperament acts as a risk or protective factor in the development of maladaptive perfectionism and related mental health outcomes.

## Figures and Tables

**Table 1:** Perfectionism levels as assessed by the Figure Copying Task (*N* = 32).

Perfectionism Levels	*N*	%
Not Perfectionist	12	37.5
Slightly Perfectionistic	8	25.0
Moderately Perfectionistic	11	34.4
Very Perfectionistic	1	3.1
Extremely Perfectionistic	0	0
Total	32	100.0

**Table 2:** Children’s perfectionism as assessed by parent-report (CAPS) and self-report (CAPS interview) measures (*N* = 32).

	Self-Report		Parent-Report		*t*	*p*	*d*	*r*	*p*
Subscales	*M*	*SD*	*M*	*SD*					
SOP	4.22	0.91	3.20	0.66	5.5	<0.001	1.28	0.14	0.452
SPP	3.33	1.24	2.57	0.61	4.0	<0.001	0.78	0.47	0.007

Note: CAPS interview scores (originally on a 1–4 scale) were rescaled to the 1–5 range to allow comparison with parent-reported CAPS; *M*: mean; *SD*: standard deviation; *t*: paired-sample t-test statistic; *p*: significance level; *d*: Cohen’s d; *r*: Pearson’s correlation; CAPS: Child and Adolescent Perfectionism Scale; SPP: Socially Prescribed Perfectionism; SOP: Self-Oriented Perfectionism.

**Table 3:** Pearson correlations of corresponding items between parent-report (CAPS) and self-report (CAPS interview) assessments of children’s perfectionism (*N* = 32).

	CAPS							
CAPS Interview	Item 1 (SOP)	Item 2 (SOP)	Item 4 (SOP)	Item 5 (SPP)	Item 8 (SPP)	Item 10 (SPP)	Item 13 (SPP)	Item 16 (S0P)
Point 1 (SOP)	−0.06	−0.15	−0.14	−0.07	0.17	−0.05	−0.10	−0.35 *
Point 2 (SOP)	0.23	−0.01	0.21	0.11	0.06	0.01	−0.11	0.02
Point 3 (SOP)	0.22	0.20	0.20	0.38 *	0.48 **	0.12	0.14	0.05
Point 4 (SPP)	0.08	0.26	0.20	0.44 **	0.53 **	0.24	−0.12	0.03
Point 5 (SPP)	0.15	0.21	−0.03	0.34 *	0.12	−0.26	−0.13	−0.09
Point 6 (SPP)	0.01	−0.01	0.04	0.57 **	0.13	−0.06	−0.28	−0.14
Point 7 (SPP)	0.27	0.25	0.23	0.54 **	0.41*	0.02	0.04	0.06
Point 8 (SOP)	0.15	−0.08	0.26	0.08	0.19	0.02	−0.10	−0.09

Note: Point 1: Do you try to be perfect in everything you do? Point 2: Do you want to be the best in everything you do? Point 3: Do you feel like you always have to do your best? Point 4: Are there people in your life who expect you to be perfect? Point 5: Does your family expect (or think you should be) perfect? Point 6: Do people expect more from you than you can actually do? Point 7: Do you think others always expect you to be perfect? Point 8: Do you always try to be as perfect as possible? Item 1: My child tries to be perfect in everything he/she does. Item 2: My child wants to be the best in everything he/she does. Item 4: My child feels he/she always has to do his/her best. Item 5: My child feels there are people in his/her life who expect him/her to be perfect. Item 8: My child feels that his/her family expects him/her to be perfect. Item 10: My child feels that people expect more from him/her than he/she is capable of. Item 13: My child feels that others always expect him/her to be perfect. Item 16: My child feels that when he/she does something, it has to be perfect. CAPS = Child and Adolescent Perfectionism Scale; SPP = Socially Prescribed Perfectionism; SOP = Self-Oriented Perfectionism * *p* < 0.05; ** *p* < 0.01.

**Table 4:** Pearson correlations between observational (Figure Copying Task), self-reported (CAPS interview) and parent-reported (CAPS) measures of children’s perfectionism (*N* = 32).

		Figure Copying Task	
		PL	No. Figures
CAPS Interview	SOP	0.34 *	0.10
	SPP	0.25	0.08
CAPS	SOP	0.02	−0.13
	SPP	0.22	−0.23

Note: CAPS: Child and Adolescent Perfectionism Scale; No. Figures: number of figures completed by the child during the Figure Copying Task; PL: perfectionism level obtained in the task; SPP: Socially Prescribed Perfectionism; SOP: Self-Oriented Perfectionism; * *p* < 0.05.

**Table 5:** Descriptive statistics and Pearson correlations between self-reported parental characteristics (HMPS and PBS) and observed behaviours (interaction task) (*N* = 32).

	HMPS		PBS		IT	
	SOP	SPP	PP	CP	IT Score	MEIT
HMPS						
SOP	1.00					
SPP	0.41 *	1.00				
PBS						
PP	−0.05	−0.50 **	1.00			
CP	0.14	0.22	−0.13	1.00		
IT						
IT score	−0.02	0.24	−0.01	0.54 ***	1.00	
MEIT (%)	−0.03	0.18	0.03	0.60 ***	0.93 ***	1.00
*M ± SD*	4.62 ± 0.69	3.05 ± 0.64	4.74 ± 0.27	2.49 ± 0.698	1.84 ± 0.39	16.67% ± 17.90

Note: HMPS: Multidimensional Perfectionism Scale; PBS: Parental Behaviour Scale; IT: interaction task; SOP: Self-Oriented Perfectionism; SPP: Socially Prescribed Perfectionism; PP: Positive Parenting subscale; CP: Coercive Parenting subscale; MEIT: Moderately or Extremely Intrusive Type; *M ± SD*: mean ± standard deviation; * *p* < 0.05; ** *p* < 0.01; *** *p* < 0.001.

**Table 6:** Pearson correlation between parental characteristics (HMPS, PBS and interaction task) and children’s perfectionism (CAPS, CAPS interview and Figure Copying Task) (*N* = 32).

	HMPS		PBS		IT	
	SOP	SPP	PP	C P	IT Score	MEIT
CAPS interview						
SOP	0.05	−0.13	0.09	0.09	−0.08	0.03
SPP	0.02	0.04	0.03	0.30	0.28	0.32
CAPS (parental report)						
SOP	0.08	0.05	−0.10	0.19	0.33	0.36 *
SPP	0.20	0.05	−0.05	0.31	0.16	0.18
Figure Copying Task						
PL	−0.06	0.01	0.02	−0.06	−0.22	−0.26
No. Figures	0.01	0.01	−0.04	0.00	0.29	0.34

Note: HMPS: Multidimensional Perfectionism Scale; PBS: Parental Behaviour Scale; IT: interaction task; SOP: Self-Oriented Perfectionism; SPP: Socially Prescribed Perfectionism; PP: Positive Parenting subscale; CP: Coercive Parenting subscale; MEIT: Moderately or Extremely Intrusive Type; CAPS: Child and Adolescent Perfectionism Scale; SOP: Self-Oriented Perfectionism; SPP: Socially Prescribed Perfectionism; PL: Perfectionism Level obtained in the task; No. Figures: number of figures completed by the child during the Figure Copying Task; * *p* < 0.05.

**Table 7:** Pearson correlations between child temperament (CBQ-SF) and children’s perfectionism (CAPS interview, CAPS and Figure Copying Task) (*N* = 32).

Dimensions CBQ-SF	CAPS Interview		Parent CAPS		Figure Copying Task	
	SOP	SPP	SOP	SPP	PL	No. Figures
Surgency/Extraversion	0.06	0.08	0.25	−0.14	−0.12	0.14
High-Intensity Pleasure	−0.09	−0.02	0.40 *	0.09	−0.07	0.05
Effortful Control	−0.13	−0.11	−0.07	−0.16	−0.17	0.08
Inhibitory Control	−0.19	−0.22	−0.11	−0.31	−0.16	−0.01
Negative Affect	−0.15	0.06	−0.06	0.21	−0.16	−0.01
Soothability	0.24	0.30	−0.07	−0.13	0.12	0.06

Note: CBQ-SF: Child Behavior Questionnaire—Short Form; CAPS: Child and Adolescent Perfectionism Scale; No. Figures: number of figures completed by the child during the Figure Copying Task; PL: perfectionism level obtained in the task; SPP: Socially Prescribed Perfectionism; SOP: Self-Oriented Perfectionism; * *p* < 0.05.

**Table 8:** Pearson correlations between child temperament (CBQ-SF) and parental behaviours/perfectionism (HMPS, PBS and interaction task) (*N* = 32).

Dimensions CBQ-SF	HMPS		PBS		IT	
	SOP	SPP	PP	CP	IT Score	MEIT
Surgency/Extraversion	0.14	0.36 *	−0.26	0.28	0.44 *	0.39 *
High-Intensity Pleasure	0.39 *	0.29	−0.38 *	0.38 *	0.37 *	0.38 *
Effortful Control	−0.14	−0.10	0.36 *	0.05	0.05	0.23
Inhibitory Control	−0.14	−0.15	0.35 *	−0.08	−0.06	0.05
Negative Affect	0.15	0.01	0.09	0.28	0.01	0.07
Shyness	−0.06	0.07	0.16	0.27	−0.01	0.04

Note: PBS: Parental Behaviour Scale; PP: Positive Parenting subscale; CP: Coercive Parenting subscale; HMPS: Multidimensional Perfectionism Scale; SPP: Socially Prescribed Perfectionism; SOP: Self-Oriented Perfectionism; IT: interaction task; MEIT: Moderately or Extremely Intrusive Type; CBQ-SF: Child Behavior Questionnaire—Short Form; * *p* < 0.05.

**Table 9:** Moderation analyses testing the role of child temperament dimensions in the association between parental and child perfectionism (*N* = 32).

Moderator	ΔR^2^	β	*p*	95% BootCI
Parent SOP → Child SOP				
Surgency/Extraversion	0.056	0.37 (0.24)	0.208	[−0.07; 1.26]
Effortful Control	0.022	−0.30 (−0.15)	0.427	[−1.07; 0.66]
Negative Affect	0.044	−0.58 (−0.27)	0.259	[−1.16; 1.50]
Parent SPP → Child SPP				
Surgency/Extraversion	0.023	0.41 (0.15)	0.419	[−0.72; 1.42]
Effortful Control	0.179 *	−1.10 * (−0.48 *)	0.020	[−2.16; −0.18]
Negative Affect	0.027	−0.77 (−0.16)	0.386	[−2.64; 1.41]

Note: ΔR^2^ = change in explained variance due to moderator; β = unstandardised regression coefficient (standardised coefficient in parentheses); *p*-value refers to the interaction term; 95% BootCI: bias-corrected bootstrapped 95% confidence interval (based on 5000 bootstrap samples); * *p* < 0.05.

## Data Availability

Data is contained within the article.

## Abbreviations

The following abbreviations are used in this manuscript:

SOP	Self-Oriented Perfectionism
SPP	Socially Prescribed Perfectionism
OOP	Other-Oriented Perfectionism
CAPS	Child and Adolescent Perfectionism Scale
SDQ	Sociodemographic Questionnaire
HMPS	Multidimensional Perfectionism Scale
PBS	Parental Behaviour Scale
PP	Positive Parenting
CP	Coercive Parenting
CBQ-SF	Children’s Behavior Questionnaire—Short Form
IT	Interaction Task
MEIT	Moderately or Extremely Intrusive Type
PL	Perfectionism Level
